# Immediate impact of hip and sacroiliac diagonal mobilization and proprioceptive exercise on Y-balance performance and force-plate postural metrics in youth male soccer players: an exploratory study

**DOI:** 10.3389/fphys.2026.1767331

**Published:** 2026-06-12

**Authors:** Paulina Hallmann, Julia Stachura, Rafał Studnicki

**Affiliations:** 1 Student Scientific Circle of Orthopedic Manual Therapy, Medical University of Gdańsk, Gdańsk, Poland; 2 Department of Physiotherapy, Medical University of Gdańsk, Gdańsk, Poland

**Keywords:** biomechanics, mobilization, postural balance, proprioception, soccer

## Abstract

This exploratory randomized crossover trial compared the immediate effects of diagonal hip/sacroiliac mobilization, a proprioceptive exercise sequence, and a sham manual-contact condition on surrogate measures of static and dynamic balance in youth male soccer players. Methods: Fifteen male soccer players aged 11–13 years completed all three conditions in randomized order. Before and immediately after each session, participants performed bipodal and single-leg K-Force force-plate tests and the Lower Quarter Y-Balance Test. The dominant-limb Y-Balance composite was treated as the principal dynamic-balance endpoint, whereas the remaining Y-Balance variables and force-plate measures were considered secondary/exploratory outcomes. Results: Most outcomes showed no consistent condition × time effect. For the secondary/exploratory bipodal force-plate battery, a nominal interaction was observed for eyes-closed right-foot center-of-pressure (COP) surface area (p = 0.021, ηp² = 0.24), reflecting greater sway after mobilization than after control (Δ = +133 mm²), a potentially destabilizing rather than beneficial change. In eyes-closed single-leg stance, COP surface asymmetry also showed a nominal interaction (p = 0.013, ηp² = 0.27); proprioceptive exercise reduced asymmetry versus control (Δ = −25.5 percentage points; p = 0.015, dz = 0.71). For the principal dynamic-balance endpoint, the dominant-limb Y-Balance composite showed a condition × time interaction (p = 0.029, ηp² = 0.22); compared with control, mobilization attenuated the post-session decline (Δ = +10.4 percentage points; p = 0.008, dz = 0.81). Conclusions: A single session of diagonal mobilization or proprioceptive exercise did not produce generalized acute improvements across the balance battery. The only between-condition signal that remained statistically significant after within-outcome adjustment was preservation of the dominant-limb Y-Balance composite after mobilization versus control. The force-plate findings were isolated exploratory results and do not support meaningful warm-up or injury-prevention claims.

## Introduction

Soccer is a globally popular team sport characterized by high-intensity intermittent efforts such as sprinting, jumping, and rapid changes of direction, which impose substantial metabolic and mechanical loads on players (Hulton et al., 2022; Sánchez et al., 2026; Shen et al., 2026). Soccer exposes youth players to a meaningful injury burden, and the lower extremity is the most frequently affected body region across youth football players (Robles-Palazón et al., 2022). Because postural control is relevant to lower-extremity function (Hrysomallis, 2007; Rodríguez et al., 2026), balance-oriented assessments are commonly used to characterize neuromuscular status in athletes, with the Lower Quarter Y-Balance Test providing a practical and reliable measure of dynamic postural control (Lesch et al., 2024). In professional male footballers (Mohammadi et al., 2024), lower and asymmetrical preseason Y-Balance scores have been associated with future lower-limb injury, but the Y-Balance Test remains a surrogate measure of dynamic postural control rather than a direct injury endpoint. Force-platform center-of-pressure variables likewise quantify postural sway during quiet and single-leg stance and are widely used to assess static postural control (Quijoux et al., 2021).

Proprioception is mediated by mechanoreceptors located in muscles, tendons, joint capsules, and skin, which signal body shape, position, movement, and muscle force to the central nervous system (Proske and Gandevia, 2012). These proprioceptive inputs are integrated with vestibular and visual information within somatosensory and sensorimotor networks to maintain postural control and regulate balance during complex whole-body tasks (Sakai et al., 2022). Proprioceptive and neuromuscular training programs can improve dynamic neuromuscular control and reduce ankle-sprain incidence in athletic populations, particularly when delivered as repeated training exposures over time (Rivera et al., 2017). Similarly, soccer-specific neuromuscular warm-up programs such as FIFA 11+ have demonstrated injury-reduction benefits when implemented repeatedly over weeks to months, so that literature supports longitudinal prevention efficacy rather than immediate effects of a single warm-up exposure (Sadigursky et al., 2017).

By contrast, the evidence that hip-directed manual therapy can acutely influence neuromuscular or balance-related outcomes is more limited and derives mainly from small studies in clinical populations rather than from trials in healthy youth athletes (Beselga et al., 2016). In patients with hip osteoarthritis, mobilization with movement has produced immediate improvements in pain, hip range of motion, and physical performance, and in patients with anterior knee pain, passive hip mobilization has acutely increased eccentric hip abductor and external rotator strength (Beselga et al., 2016). These findings suggest that hip mobilization can induce short-term physiological responses, but they do not by themselves establish that a single manual therapy session will meaningfully enhance balance in youth athletes.

Diagonal or multiplanar mobilization approaches, rooted in proprioceptive neuromuscular facilitation concepts, aim to stimulate complex capsular and periarticular mechanoreceptors through three-dimensional patterns that more closely reflect functional movement demands and have been reported to improve balance and gait parameters in neurological populations (Boob and Kovela, 2022). Extending these concepts to sport, a recent randomized double-blind trial in young soccer players reported that a single session of diagonal mobilization led to acute improvements in static and dynamic balance derived from force-plate metrics, suggesting that such manual interventions can modulate postural control in trained youth athletes (Studnicki et al., 2025). Nevertheless, contemporary literature on youth soccer injury prevention emphasizes neuromuscular exercise and screening but provides limited evidence directly comparing the acute effects of diagonal hip mobilization versus structured proprioceptive training on balance and force-platform–based postural outcomes (Read et al., 2018).

In elite male youth soccer players, maturation-related changes in neuromuscular function and dynamic balance, as captured by anterior-reach Y-Balance performance and prospective injury data, have been associated with injury risk, highlighting the importance of age-appropriate screening and targeted interventions during early adolescence (Read et al., 2020). Despite the widespread use of Y-Balance testing and force-platform measures to profile dynamic and static postural control in youth soccer, there is a paucity of studies evaluating how single-session manual therapy and proprioceptive exercise interventions differentially influence these kinetic and kinematic parameters in youth players (González-Fernández et al., 2022). Therefore, the aim of the present randomized crossover study was to compare the immediate effects of diagonal hip and sacroiliac mobilization, a standardized proprioceptive exercise sequence, and a sham manual-contact condition on dynamic balance assessed with the Lower Quarter Y-Balance Test and on static postural-control variables derived from force-platform center-of-pressure recordings in competitive male soccer players aged 11 to 13 years. A secondary aim was to examine whether either active intervention produced more favorable short-term changes in weight-distribution or asymmetry-related balance measures than sham, while restricting interpretation to acute surrogate outcomes rather than injury-prevention effects.

## Methods

### Trial design and setting

This exploratory study was a single-centre, randomized, crossover, three-arm trial with repeated pre–post measurements, conducted in a youth soccer academy context. Because crossover trials are vulnerable to period, sequence, and carryover effects, treatment effects were evaluated with explicit consideration of these design features rather than by treating the dataset as a simple repeated-measures structure alone. Fifteen male outfield players aged 11–13 years, all members of the same competitive team at Escola Futbolu in Pruszcz Gdański (Poland), were randomly allocated in a 1:1:1 ratio to one of three intervention conditions: diagonal mobilization of the hip and sacroiliac joints, a standardized proprioceptive exercise program, or a placebo manual intervention. The trial was designed within an exploratory superiority framework to compare the immediate effects of these three physiotherapeutic approaches on dynamic balance (Y-Balance Test) and force-platform–derived postural control parameters.

All interventions were delivered as a single session embedded within the players’ regular in-season training microcycle. For each athlete, outcome measures were collected immediately before and immediately after the assigned intervention during the same visit, with no longer-term follow-up assessments planned. The study was organised over three consecutive weeks (nine assessment days in total), with sessions held on Monday, Wednesday, and Friday at the training facility; on each study day all 15 players were assessed, and group allocation determined which intervention they received. Assessments were performed at two measurement stations (Y-Balance Test and K-Force force platform), with players rotating between stations according to a predefined schedule to minimize waiting time and to maintain similar testing conditions across interventions.

The trial was conducted in accordance with the principles of the Declaration of Helsinki and with local regulations governing research involving minors. Ethical approval was obtained from the Independent Bioethics Committee for Scientific Research at the Medical University of Gdańsk (Resolution No. KB 523/2025, 20 November 2025), and the study was authorised by the management of Escola Futbolu in Pruszcz Gdański. Before any study procedures were initiated, all participants and their parents or legal guardians received a detailed verbal and written explanation of the study protocol, and written informed consent was obtained from a parent or legal guardian. Players provided age-appropriate verbal assent.

The trial protocol, including the eligibility criteria, intervention procedures, outcome measures, and statistical analysis plan, was finalised before recruitment commenced and approved by the Independent Bioethics Committee for Scientific Research at the Medical University of Gdańsk. No changes were made to the trial design, interventions, or pre-specified outcomes after the start of participant enrolment.

### Participants

Participants were recruited using a convenience sampling strategy. All outfield players from a single under-13 competitive team were approached during scheduled meetings with players and their parents at the training facility and were invited to be screened for eligibility.

Eligibility criteria were defined *a priori*. Inclusion criteria were: (i) age between 11 and 13 years; (ii) regular participation in organised soccer training with the study team; and (iii) availability to attend all scheduled testing sessions and to complete both pre- and post-intervention assessments on the designated study days. Exclusion criteria were: (i) history of surgery or musculoskeletal injury involving the hips, knees, ankles, or lumbar spine in the previous 6 months; (ii) current pain in any lower-limb joint (hip, knee, or ankle); (iii) clinically evident lower-limb joint hypermobility; and (iv) any known neurological or connective tissue disorder that could affect balance, postural control, or safe participation in training and testing.

A total of 47 players were initially considered (all outfield players from the relevant age categories in the club). After screening, 30 were excluded because they did not meet the age criterion (outside 11–13 years), and 2 were excluded due to a recent lower-limb injury in the month preceding data collection. The final sample therefore comprised 15 male youth soccer players who met all inclusion criteria, none of the exclusion criteria, and provided written informed assent in addition to parental or guardian consent.

At baseline, the included players had a mean (SD) age of 12.7 (0.5) years, a mean height of 161.7 (5.4) cm, and a mean body mass of 49.8 (5.2) kg. Mean lower-limb length was 87.7 (5.4) cm for both the left and right sides, with no measurable leg-length discrepancy in any participant. These anthropometric data were collected by a trained researcher using standardised measurement procedures and recorded in a dedicated database prior to randomisation.

All participants were active competitive players training three times per week under the supervision of club coaching staff. Typical training sessions lasted 80–90 minutes and followed a structured format consisting of a general warm-up (general development exercises), followed by individual technical drills and team tactical exercises. No structured proprioceptive training program and no routine diagonal hip or sacroiliac joint mobilization were part of the regular training schedule before the start of the trial, and all players were cleared for full participation by the club’s coaching and medical staff at enrolment.

### Interventions and comparators

This was a randomized crossover trial in which all 15 participants completed three separate intervention sessions, each corresponding to one of the study conditions: (1) diagonal mobilization of the hip and sacroiliac joints (DM), (2) a supervised proprioceptive exercise program (PE), and (3) a placebo mobilization (PL). Each intervention was administered on a separate assessment day within the in-season training period, with at least 48 hours between sessions (Monday–Wednesday–Friday schedule), and the same standardized pre–post testing battery (Y-Balance Test and K-Force force platform assessments) was applied immediately before and after each intervention. All sessions were conducted in a quiet indoor room at the training facility of Escola Futbolu in Pruszcz Gdański, under stable environmental conditions, and were embedded in the athletes’ regular weekly microcycle without altering their usual team training content. The same licensed physiotherapist (25 years’ experience in orthopedic manual therapy and sports physiotherapy) delivered all manual interventions and supervised the exercise program to ensure protocol fidelity and consistency across participants and conditions.

Across the three intervention weeks, each player attended three intervention days and thus received each of the three protocols once, always in conjunction with the identical pre–post assessment procedures. The duration of the DM and PL sessions was 8 minutes per participant, whereas the PE protocol lasted approximately 16 minutes. No co-interventions were introduced by the study team between pre- and post-measurements on a given day, and there were no changes to the players’ usual training between intervention days other than the study procedures themselves. Adherence at the session level was 100%, as all participants completed the full dose of each assigned intervention without early termination or protocol deviation.

The three conditions were not time-matched: diagonal mobilization and sham manual contact lasted approximately 8 min, whereas the proprioceptive exercise condition lasted approximately 16 min. This difference reflects the practical structure of the exercise sequence used in the study. However, it also means that any between-condition differences involving the proprioceptive condition may reflect not only intervention content but also unequal exposure duration, therapist attention, or exercise-induced arousal.

### Diagonal hip and sacroiliac mobilization (DM group)

Participants allocated to the DM group received a single session of bilateral diagonal mobilization of the hip and sacroiliac (SI) joints. The intervention consisted of passive, rhythmic sliding mobilizations applied in three-dimensional directions with the goal of providing an intense proprioceptive and sensorimotor stimulus to structures around the hip and pelvic girdle. Each participant first underwent pre-intervention assessment, then received the mobilization protocol, and finally underwent post-intervention assessment during the same visit. For the hip joint component, participants lay prone on a plinth with their arms alongside the body and the feet relaxed. The therapist positioned both hands over the proximal femur and applied oscillatory mobilizations combining medial, cranial, and ventral directions, producing a diagonally oriented glide within a comfortable mid-range of motion. Mobilization was performed first on one side and then on the contralateral side. For the SI joint component, the therapist placed the hands over the sacrum and applied rhythmic, low-velocity mobilizations combining medial, ventral, and cranial directions, again in a diagonal pattern designed to stimulate capsular and periarticular mechanoreceptors. All mobilizations were performed within the “second stage of mobilization” as defined in the study protocol, i.e., non-thrust, non-painful mobilizations not reaching the end-range barrier.

On each side, the hip mobilization was applied for 2 minutes and the SI mobilization for 2 minutes, resulting in a total treatment time of 8 minutes per participant (4 minutes per side). The sequence (hip then SI on one side, followed by hip then SI on the opposite side) and rhythm of oscillations were standardized and rehearsed before data collection. The intensity of mobilization was adjusted to remain painless and comfortable while maintaining consistent amplitude and frequency across participants, as monitored by the treating physiotherapist. No individual adjustment or progression was used because the study focused on the acute response to a single standardized mobilization session. All participants in this group completed the full 8-minute protocol without adverse events or early termination.

### Proprioceptive exercise program (PE group)

Participants in the PE group received a single session of a supervised, multicomponent proprioceptive and balance exercise program performed in the same indoor space and under the direct supervision of the study physiotherapist. Exercises were performed individually, with continuous verbal feedback on posture and movement quality. The program was designed to provide an approximately 16-minute neuromuscular stimulus targeting single-leg and double-leg balance, postural control on unstable surfaces, and rapid transitions between stable and unstable tasks. All exercises were performed in athletic footwear on standard gym flooring, with additional use of foam pads, sensory discs, a gymnastic stick, and small plastic “mushroom” markers. The sequence and dosage of the eight exercises were fixed for all participants and sessions: (1) single-leg half-squat on stable ground for 1 minute on each lower limb (2 minutes total); (2) double-leg jumps with 90° rotations around the body’s vertical axis on a foam mat for 2 minutes, with change in rotation direction after 1 minute; (3) single-leg stance on a sensory disc while bouncing a ball with the free lower limb for 2 minutes; (4) double-leg stance on two sensory discs while following the end of a gymnastic stick with the index finger, guided by a researcher, for 2 minutes with change of guiding hand after 1 minute; (5) single-leg stance on a disc while performing “skip A” movements (high-knee march) with the free limb for 2 minutes, switching the stance limb after 1 minute; (6) single-leg stance with slight hip and knee flexion while the ipsilateral upper limb provided lateral trunk support and the contralateral upper limb reached to touch color-marked “mushrooms” placed in front, behind, and to both sides for 2 minutes, switching sides after 1 minute; (7) single-leg stance while moving a “mushroom” under the stance foot using the free foot for 2 minutes, with leg change after 1 minute; and (8) repeated 3-second on-the-spot sprints followed by a single-leg jump onto a foam pad, alternating legs, for 2 minutes.

Participants were instructed to perform all tasks with controlled, sport-specific movement quality, to maintain balance as long as possible, and to resume the exercise immediately after any loss of balance. The physiotherapist provided standardized cues (e.g., “keep your trunk upright,” “look straight ahead,” “control the landing”) but did not progress or regress exercises, to avoid inter-individual variation in stimulus dosage. No home program was prescribed, and no additional balance training was allowed on the day of testing. All participants assigned to the PE group completed the full set of exercises with no reporting of pain, dizziness, or other adverse events.

### Placebo mobilization (PL group – comparator)

The PL group served as a manual-contact comparator to control for non-specific effects such as touch, therapist–patient interaction, and time spent on the treatment table, without delivering therapeutic joint mobilization. Participants in this group underwent a sham procedure in which the physiotherapist placed the hands on the same anatomical regions used in the DM protocol (proximal femur and sacral area) while the participant lay prone on the plinth. The therapist applied gentle, static, non-directional pressure to the soft tissues at a clearly subtherapeutic intensity, deliberately avoiding any oscillatory movement, joint gliding, traction, or end-range techniques at the hip or SI joints.

The spatial arrangement of the therapist’s hands, participant positioning, communication style, and total contact time were matched to the DM protocol. Specifically, gentle static contact was maintained for 2 minutes over the hip region and 2 minutes over the sacral region on each side, for a total of 8 minutes per participant. Participants were informed that they were receiving a manual therapy intervention but were not told whether it was active or placebo. No additional exercises, mobilizations, or stretching were performed during this period. All participants assigned to the PL group tolerated the procedure well, reported no discomfort, and completed the full 8-minute session.

Across all three groups, there were no protocol deviations, and all participants completed their assigned intervention and both pre- and post-intervention assessments at each study visit, ensuring comparable exposure time and measurement conditions between the active interventions and the comparator.

### Assessment procedures

All assessments were conducted in an indoor facility at the team’s training center, under stable environmental conditions and at approximately the same time of day for each player across all sessions to minimize diurnal variation. Before each experimental session, athletes completed a standardized 10-min warm-up consisting of low-intensity running and mobility exercises, followed by pre-intervention testing, the assigned intervention, and post-intervention testing using the same test order for that player. A standardized warm-up was applied before each session to reduce between-session procedural variability and to reflect the pre-activity context in which the interventions would be used. Nevertheless, because warm-up itself can acutely influence postural-control outcomes, the present design evaluates the incremental effect of each intervention on top of a common warm-up, rather than the isolated effect of the intervention in a non-warmed state.

Dynamic balance was assessed with the Lower Quarter Y-Balance Test (YBT-LQ), and static and single-leg postural control were assessed on a dual portable force-platform system (K-Force, Kinvent, Montpellier, France). Players were instructed to avoid strenuous exercise and caffeine for 24 h before testing and to maintain their usual diet and hydration. All tests were administered by the same two experienced physiotherapists, who provided standardized instructions and verbal encouragement and were not involved in subsequent data analysis.

For feasibility reasons, a single eyes-open and a single eyes-closed single-leg force-plate trial were recorded per limb at each time point. Because single-trial COP estimates are more vulnerable to within-subject variability than averages derived from repeated trials, these single-leg force-plate outcomes were interpreted as lower-precision/exploratory measures.

### Y-balance test (dynamic balance)

Dynamic balance was evaluated using the YBT-LQ on a commercial apparatus with three reach indicators oriented in the anterior, posteromedial, and posterolateral directions, separated by 135° between the posterior arms. The YBT-LQ is a modified version of the Star Excursion Balance Test and has been shown in a systematic review and meta-analysis to present good to excellent intra- and inter-rater reliability, as well as discriminant and injury-prediction validity in youth and adult athletic populations (Plisky et al., 2021). Further work has confirmed excellent inter-rater reliability (ICC 0.99–1.00) and construct validity of the YBT-LQ in young adults, supporting its use as a robust measure of dynamic balance in both clinical and sports contexts (Alshehre et al., 2021).

For testing, players wore their usual indoor athletic footwear and stood on the central footplate with the great toe of the stance foot aligned to the front edge of the platform, hands placed on the hips, and eyes directed forward. The dominant limb (defined as the preferred kicking leg) and non-dominant limb were tested separately. Before recorded trials, each athlete performed one full familiarization sequence of reaches in the three directions on each limb under supervision. After familiarization, three test trials were performed in the following order of reach directions: anterior, posteromedial, and posterolateral, with 10–15 s of rest between trials within a direction and approximately 45–60 s between directions. Any trial in which the athlete lost balance, removed the stance foot from the platform, failed to return the reaching leg to the starting position under control, or used the reaching limb for weight bearing on the floor or indicator was discarded and immediately repeated according to standard YBT procedures (Plisky et al., 2021).

Reach distances were recorded to the nearest 0.5 cm and subsequently normalized to limb length (reach distance/limb length × 100), where limb length was measured as the distance from the anterior superior iliac spine to the distal tip of the medial malleolus for each leg (Plisky et al., 2021). For each direction and limb, the maximum successful normalized reach distance across the three trials was retained. A normalized composite reach score (%) was calculated as the sum of the maximal normalized reach distances in the three directions divided by three, which is the most commonly used summary index of dynamic balance in the YBT-LQ literature (Plisky et al., 2021). The primary dynamic balance outcome for this study was the normalized composite reach score of the dominant limb, while direction-specific normalized maximal reach distances in each of the three directions for both limbs were treated as secondary dynamic balance outcomes.

### Force-platform assessments

Static and single-leg postural control were quantified using a pair of portable K-Force plates connected via Bluetooth to the manufacturer’s proprietary application, which computes vertical ground-reaction forces and centre-of-pressure (COP)–based summary metrics. Portable Kinvent plates have demonstrated good validity and reliability for static and dynamic balance tasks when compared with laboratory-grade force platforms, supporting their use in field-based assessments of postural control (Meras Serrano et al., 2023). More broadly, COP-derived variables measured on force platforms during quiet standing (bipodal and unipedal) show good to excellent test–retest reliability in children, adolescents, and adults (Barozzi et al., 2014; Lo et al., 2022).

For each K-Force test condition, players stood barefoot on the plates and were instructed to remain as still as possible, breathe normally, and fix their gaze on a point at eye level on the opposite wall. Trials were recorded for a fixed duration pre-set in the K-Force software (identical for all conditions and sessions), and the same sampling frequency and processing parameters were used throughout according to the device default settings. Each condition (bipodal EO, bipodal EC, single-leg EO, single-leg EC) was recorded once per player per time point (pre- and post-intervention). Between conditions, athletes were allowed a brief standing or seated rest (approximately 30–60 seconds) to minimise fatigue and maintain attention. Trials with obvious loss of balance, stepping, or technical artefacts (e.g. sensor dropout) were immediately repeated. Only trials judged as valid by the examiners were exported. From the K-Force software, we exported only the summary variables that appear in the datasets: percentages of weight distribution and COP surface-related measures for each foot or foot segment and their asymmetry indices.

### Bipodal stance (static postural control)

Bipodal stance was assessed with each foot placed centrally on a separate K-Force plate, feet approximately shoulder-width apart, knees extended but unlocked, arms relaxed alongside the body, and weight distributed comfortably between limbs. Two sensory conditions were tested: EO, with players focusing on a fixed point on the wall, and EC, with visual input occluded immediately before the trial while maintaining the same stance. For each intervention day and time point (pre- and post-intervention), one valid EO trial and one valid EC trial were recorded, and data were exported. From these bipodal trials, the following outcomes were collected and are present in the spreadsheets for each condition (before/after, EO/EC) and each player: (i) Right and left weight distribution (%), expressed as the percentage of total vertical force borne by the right and left plates, respectively, as indices of inter-limb load symmetry; (ii) Right and left foot surface of COP (mm²), representing the COP surface area under each foot separately, as measures of sway magnitude at each limb; and (iii) Asymmetry of surface of COP (%), defined by the K-Force software as the relative difference in COP surface area between the two feet, reflecting asymmetry in postural sway between limbs. These three variables (right/left weight distribution, right/left COP surface, COP surface asymmetry) constituted the primary static postural control outcomes for bipodal stance in both EO and EC conditions.

### Single-leg stance (unipedal postural control and antero-posterior load distribution)

Single-leg stance was assessed with the K-Force plates configured according to the manufacturer’s recommendations for evaluating unilateral support and antero-posterior loading strategy. For each condition, stance on the right and left lower limbs was tested separately, both with EO and EC, following the same general instructions regarding posture and gaze. Each player performed one valid EO trial and one valid EC trial in single-leg stance on each limb at each time point (pre- and post-intervention), and data were exported sheets. Force-platform measures of unipedal COP displacement are commonly used to assess neuromuscular control and ankle–hip strategy in athletes and have shown acceptable reliability in team-sport populations (Troester et al., 2018; Meras Serrano et al., 2023). From these single-leg stance trials, the following outcomes were collected and appear in the datasets for each player, limb, and condition: (i) Right and left foot surface of COP (mm²), representing COP surface area estimated separately for each plate under the stance foot, as indices of sway magnitude; (ii) Asymmetry of surface of COP (%), expressing the relative difference between COP surface areas computed for the two plates during single-leg stance, used here as a measure of within-foot or medio-lateral sway asymmetry depending on foot–plate contact configuration; (iii) Right and left weight distribution – heel (%), indicating the proportion of vertical force borne by the posterior part of the stance foot (rear half); and (iv) Right and left weight distribution – top of the feet (%), indicating the proportion of vertical force borne by the anterior part of the stance foot (forefoot). Thus, for single-leg stance we focused on COP surface metrics (per-plate surfaces and their asymmetry) and antero-posterior weight-distribution indices (heel vs top of the foot) as primary unipedal postural control outcomes.

### Randomization and blinding

Randomization was applied to the sequence of exposure rather than to group membership. Three intervention sequences (A, B, C) were defined *a priori* so that each intervention appeared once in each of the three periods (Latin-square structure). Participants were then randomly assigned in a 1:1:1 ratio to one of these three sequences, which determined the order in which they received the three interventions across the three experimental days. The random allocation sequence was generated using opaque envelopes by an investigator who was not involved in intervention delivery, outcome assessment, or data analysis. A schedule linking participant identification numbers to intervention sequences (A, B, or C) was created and stored in a secure file. After baseline eligibility assessment and anthropometric measurements, each participant was assigned the next available sequence on this list. The treating physiotherapist used this schedule to know which intervention to deliver at each of the three sessions for each player, whereas outcome assessors had access only to participant codes and session numbers (1, 2, or 3), not to the underlying intervention sequence.

Two members of the research team were responsible for screening, obtaining consent/assent, and enrolling participants; they had no role in generating the randomization list. The randomization list and sequence allocations were prepared by a separate investigator. Once a player was enrolled, he was assigned the next sequence on the list, and this sequence determined which intervention he received on each of the three measurement days (with at least 48 hours between sessions). The treating physiotherapist, who delivered all three intervention types, was informed of the intervention scheduled for each player on a given day only when planning that specific session. Outcome assessors and the statistician did not have access to the allocation list.

Because of the hands-on nature of the procedures, the treating therapist was not blinded to condition allocation. The sham condition was intended to mimic therapist contact without delivering the active mobilization manoeuvre, however, participant blinding credibility and treatment expectancy were not formally assessed after each session. Participants and their parents were informed that the study compared three different physiotherapy-based warm-up approaches, but they were not told which of the three was considered a placebo or which was hypothesized to be more effective. The placebo mobilization was designed to mimic the body positioning, therapist contact, and session duration of the active diagonal mobilization while omitting any joint-gliding or oscillatory forces, in order to enhance participant blinding with respect to the manual interventions.

Outcome assessors who administered the Y-Balance Test and K-Force plate measurements were blinded to the intervention performed in each session. For them, each test session was identified only by a participant code and session number. Data exported from the Y-Balance and K-Force software were coded without labels indicating the intervention type, and the primary statistical analyses were conducted by a statistician who remained blinded to the identity of the interventions until all primary analyses had been completed.

### Sample size

The calculation was based on the condition × time interaction for the dominant-limb Lower Quarter Y-Balance Test composite score in a 2 × 3 repeated-measures design (three within-subject conditions, each assessed pre- and post-intervention), rather than on a simple paired comparison. Because a validated minimally important acute change for the Y-Balance composite in 11–13-year-old soccer players is not established, the planning assumptions were anchored to published measurement properties and to the modest acute effects reported in prior single-session intervention studies (Schwiertz et al., 2019; Harry-Leite et al., 2022; Studnicki et al., 2025). The Y-Balance Test shows good test–retest reliability in adolescents and active populations (ICC approximately 0.79–0.86), whereas minimal detectable change values suggest that changes smaller than about 5% for the composite score, and approximately 4.9–16.1% across adolescent outcomes, may reflect measurement error rather than a true performance shift (Schwiertz et al., 2019; Harry-Leite et al., 2022; Studnicki et al., 2025). We therefore assumed a conservative moderate interaction effect (Cohen’s f = 0.25; approximately ηp² = 0.06), with α = 0.05, power = 0.80, six repeated measurements, an average within-subject correlation of 0.75, and a nonsphericity correction of ϵ = 0.75. Under these assumptions, approximately 24–28 participants would have been required; allowing for attrition, a target sample of about 27–30 players would have been more appropriate. However, due to exploratory nature of our study, we have the present sample of 15 players which should be interpreted as exploratory and likely underpowered to detect small acute effects.

### Statistical procedures

All 15 participants contributed complete pre- and post-intervention data for the three within-subject conditions (diagonal mobilization, proprioceptive exercise, and control). To improve auditability, outcomes were organized hierarchically before re-analysis. The prespecified primary efficacy endpoint was the normalized Y-Balance composite score of the dominant limb. The non-dominant composite score, direction-specific Y-Balance reaches, bipodal force-plate metrics, and single-leg force-plate metrics were treated as secondary outcomes. **Table 1** lists the endpoint hierarchy and the operational definitions used in the revised analysis.

**Table 1:** Endpoint hierarchy and definitions used in the analysis.

Tier	Endpoint set	Definition
Primary	Y-Balance dominant-limb composite score (%)	[(Anterior + posteromedial + posterolateral reach)/3] ÷ limb length × 100. The right limb was analysed as the dominant limb in the original protocol.
Secondary	Y-Balance non-dominant composite and direction-specific reaches (%)	Maximum successful reach in each direction, normalized to limb length as reach ÷ limb length × 100.
Secondary	Bipodal EO and EC weight distribution (%)	Percentage of total vertical load borne by the right and left plates during quiet standing.
Secondary	Bipodal EO and EC COP surface (mm²)	Center-of-pressure surface area under the right and left feet.
Secondary	Bipodal EO and EC COP asymmetry (%)	Exported asymmetry values were numerically consistent with |larger COP surface − smaller COP surface| ÷ larger COP surface × 100.
Secondary	Single-leg EO and EC COP surface and COP asymmetry	Sway area variables from unilateral stance. COP is device-derived asymmetry index during single-leg stance.
Secondary	Single-leg EO and EC heel and forefoot load (%)	Percentage of vertical load borne by the posterior (heel) and anterior (forefoot) parts of the stance foot; the two variables are complementary and sum approximately to 100%.

COP, center of pressure; EO, eyes open; EC, eyes closed.

For each endpoint, the intervention effect was tested on the within-subject change score (Δ = post − pre) using a one-factor repeated-measures ANOVA with condition (three levels) as the within-subject factor. This formulation is algebraically equivalent to the condition × time interaction from the original 3 × 2 repeated-measures model, but it yields a more concise estimate of the acute intervention effect and avoids unnecessary emphasis on baseline comparisons in the main text. When an omnibus condition effect on change scores was detected (p < 0.05), paired *post hoc* comparisons of change scores were performed for diagonal mobilization versus control, proprioceptive exercise versus control, and diagonal mobilization versus proprioceptive exercise, with Holm adjustment within endpoint.

To address the reviewers’ concern about multiplicity across the endpoint battery, Holm’s step-down procedure was additionally applied across all 32 endpoint-level omnibus tests. Unadjusted p values are reported for transparency, but inferential emphasis is placed on the battery-wise Holm-adjusted results for the full endpoint set. Effect sizes are reported as partial eta-squared (ηp²) for omnibus repeated-measures effects and Cohen’s dz for paired contrasts. Alongside p values, the revised results present mean changes and 95% confidence intervals so that statistical significance is interpreted together with precision and magnitude.

For the COP asymmetry variables, the exported values were numerically consistent with |larger value − smaller value| ÷ larger value × 100. Given the absence of protocol-specific SEM/MDC estimates for these proprietary K-Force summaries in the present sample, isolated nominally significant force-plate findings were interpreted conservatively and not treated as clinically or practically definitive unless they were internally consistent across related endpoints.

All tests were two-sided with α = 0.05. Analysis was performed in Python using statsmodels and SciPy.

## Results

All 15 players completed all three conditions and both time points. Therefore, the repeated-measures analyses were based on a complete balanced dataset. **Table 2** summarizes the primary endpoint and the secondary outcomes that showed nominal omnibus signals before battery-wise multiplicity correction, whereas **Table 3** provides the full endpoint battery. Across the 32 endpoint-level tests, none remained statistically significant after Holm adjustment across the full battery.

**Table 2:** Primary endpoint and nominal secondary signals from the analysis.

Tier	Outcome	Diagonal mobilization Δ (95% CI)	Proprioceptive exercise Δ (95% CI)	Control Δ (95% CI)	F(2,28)	p	Holm-adjusted p across 32 endpoints	ηp²
Primary	dominant composite (%)	2.1 (-2.6 to 6.8)	3.4 (-5.1 to 11.9)	-8.3 (-15.4 to -1.3)	4.01	0.029	0.793	0.223
Secondary	non-dominant composite (%)	1.1 (-3.0 to 5.2)	4.0 (-4.1 to 12.1)	-5.6 (-13.1 to 1.9)	2.10	0.141	1.000	0.131
Secondary	dominant anterior (%)	-1.6 (-6.0 to 2.8)	2.2 (-1.3 to 5.6)	-6.7 (-11.8 to -1.6)	4.28	0.024	0.697	0.234
Secondary	EC right COP surface (mm²)	122.3 (17.6 to 227.0)	5.3 (-54.8 to 65.3)	-11.2 (-71.5 to 49.0)	4.45	0.021	0.650	0.241
Secondary	EC COP surface asymmetry (%)	1.6 (-8.6 to 11.8)	-19.9 (-36.2 to -3.6)	5.6 (-5.0 to 16.2)	5.05	0.013	0.428	0.265
Secondary	EC left heel load (%)	2.7 (-1.6 to 6.9)	3.4 (-0.1 to 6.9)	-3.1 (-6.4 to 0.1)	4.32	0.023	0.697	0.236
Secondary	EC left forefoot load (%)	-2.7 (-6.9 to 1.6)	-3.4 (-6.9 to 0.1)	3.1 (-0.1 to 6.4)	4.32	0.023	0.697	0.236

Values are mean change (post – pre) with 95% confidence intervals unless stated otherwise. The Holm-adjusted column applies the reviewer-requested family-wise correction across all 32 endpoint-level omnibus tests. None of the listed outcomes remained significant after this correction.

**Table 3:** Full endpoint for the 32 omnibus repeated-measures tests on change scores.

Tier	Domain	Outcome	F_(2,28)_	Unadjusted p	Holm p	ηp²
Primary	Y-Balance	dominant composite (%)	4.01	0.029	0.793	0.223
Secondary	Y-Balance	non-dominant composite (%)	2.10	0.141	1.000	0.131
Secondary	Y-Balance	dominant anterior (%)	4.28	0.024	0.697	0.234
Secondary	Y-Balance	dominant posteromedial (%)	2.51	0.099	1.000	0.152
Secondary	Y-Balance	dominant posterolateral (%)	2.82	0.076	1.000	0.168
Secondary	Y-Balance	non-dominant anterior (%)	2.05	0.147	1.000	0.128
Secondary	Y-Balance	non-dominant posteromedial (%)	2.01	0.152	1.000	0.126
Secondary	Y-Balance	non-dominant posterolateral (%)	1.00	0.382	1.000	0.066
Secondary	Bipodal	Bipodal EO right weight distribution (%)	2.20	0.130	1.000	0.136
Secondary	Bipodal	Bipodal EO left weight distribution (%)	2.20	0.130	1.000	0.136
Secondary	Bipodal	Bipodal EO right COP surface (mm²)	1.07	0.356	1.000	0.071
Secondary	Bipodal	Bipodal EO left COP surface (mm²)	1.07	0.358	1.000	0.071
Secondary	Bipodal	Bipodal EO COP surface asymmetry (%)	0.50	0.611	1.000	0.035
Secondary	Bipodal	Bipodal EC right weight distribution (%)	1.81	0.183	1.000	0.114
Secondary	Bipodal	Bipodal EC left weight distribution (%)	1.81	0.183	1.000	0.114
Secondary	Bipodal	Bipodal EC right COP surface (mm²)	4.45	0.021	0.650	0.241
Secondary	Bipodal	Bipodal EC left COP surface (mm²)	1.09	0.349	1.000	0.072
Secondary	Bipodal	Bipodal EC COP surface asymmetry (%)	0.45	0.645	1.000	0.031
Secondary	Single-leg	Single-leg EO right COP surface (mm²)	0.80	0.457	1.000	0.054
Secondary	Single-leg	Single-leg EO left COP surface (mm²)	1.89	0.170	1.000	0.119
Secondary	Single-leg	Single-leg EO COP surface asymmetry (%)	0.64	0.532	1.000	0.044
Secondary	Single-leg	Single-leg EO right heel load (%)	0.69	0.509	1.000	0.047
Secondary	Single-leg	Single-leg EO right forefoot load (%)	1.35	0.276	1.000	0.088
Secondary	Single-leg	Single-leg EO left heel load (%)	0.92	0.410	1.000	0.062
Secondary	Single-leg	Single-leg EO left forefoot load (%)	1.05	0.364	1.000	0.070
Secondary	Single-leg	Single-leg EC right COP surface (mm²)	3.33	0.050	1.000	0.192
Secondary	Single-leg	Single-leg EC left COP surface (mm²)	1.87	0.172	1.000	0.118
Secondary	Single-leg	Single-leg EC COP surface asymmetry (%)	5.05	0.013	0.428	0.265
Secondary	Single-leg	Single-leg EC right heel load (%)	1.41	0.261	1.000	0.091
Secondary	Single-leg	Single-leg EC right forefoot load (%)	1.41	0.260	1.000	0.092
Secondary	Single-leg	Single-leg EC left heel load (%)	4.32	0.023	0.697	0.236
Secondary	Single-leg	Single-leg EC left forefoot load (%)	4.32	0.023	0.697	0.236

The omnibus test is the repeated-measures ANOVA on within-subject change scores (post − pre) across the three conditions; this is algebraically equivalent to the condition × time interaction from the original 3 × 2 repeated-measures ANOVA.

**Figure 1:**
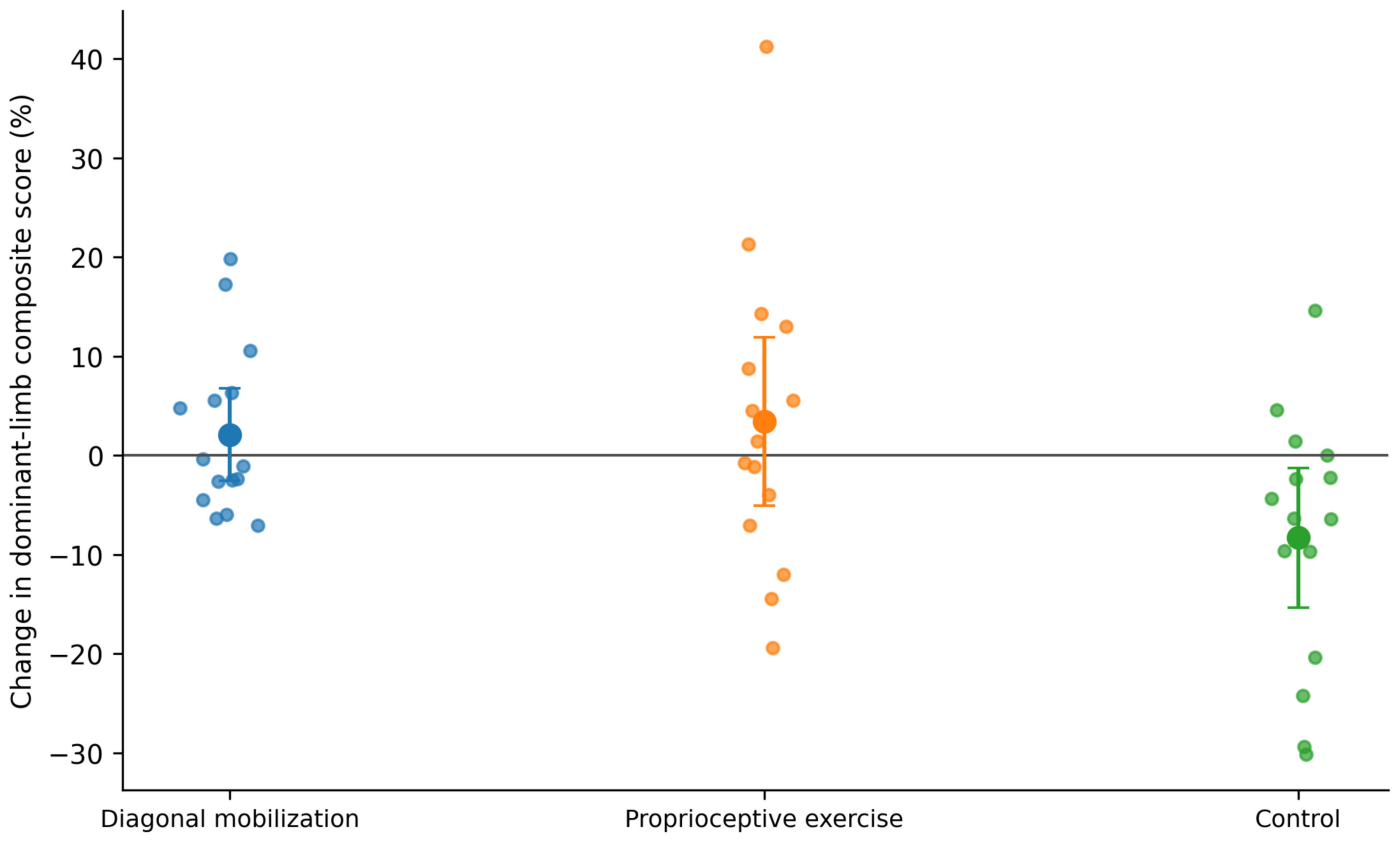
Change in the primary endpoint (dominant-limb Y-Balance composite score) by condition. Points denote individual within-subject change scores; larger filled markers with whiskers denote mean change and 95% confidence intervals. Positive values indicate post-intervention improvement and negative values indicate decline from pre-intervention.

The prespecified primary endpoint, the dominant-limb Y-Balance composite score, showed a nominal condition effect on change scores (F_(2,28)_ = 4.01, p = 0.029, ηp² = 0.223). Mean change was +2.1 percentage points (95% CI −2.4 to 6.6) after diagonal mobilization, +3.4 (−5.1 to 11.9) after proprioceptive exercise, and −8.3 (−15.4 to −1.2) after control. In the within-endpoint *post hoc* analysis, diagonal mobilization differed from control by +10.4 percentage points (95% CI 3.3 to 17.6; Holm-adjusted p = 0.023; dz = 0.81), whereas the proprioceptive exercise versus control contrast was less precise (+11.7 points, 95% CI 0.6 to 22.9; Holm-adjusted p = 0.080; dz = 0.58). However, the battery-wise Holm-adjusted p value for the primary endpoint was 0.793, indicating that this apparent signal was not robust when interpreted against the full multiplicity burden. **Figure 1** shows the individual and mean change scores for the primary endpoint.

The remaining Y-Balance outcomes did not provide convergent support for a treatment effect. The non-dominant composite score showed no condition effect on change scores (F_(2,28)_ = 2.10, p = 0.141, ηp² = 0.131). Among the direction-specific measures, only dominant-limb anterior reach showed a nominal omnibus effect (F(2,28) = 4.28, p = 0.024, ηp² = 0.234), driven mainly by a smaller decline after proprioceptive exercise than after control (mean difference in change = +8.9 percentage points, 95% CI 1.1 to 16.6; within-endpoint Holm-adjusted p = 0.083). This signal also failed to survive battery-wise correction (Holm-adjusted p = 0.697). No other direction-specific reach outcome showed evidence of a condition effect.

For the force-plate outcomes, the pattern was similarly isolated and inconsistent. In bipodal stance, only the eyes-closed right-foot COP surface showed a nominal condition effect on change scores (F_(2,28)_ = 4.45, p = 0.021, ηp² = 0.241). Mean change was +122.3 mm² (95% CI 31.9 to 212.7) after diagonal mobilization, +5.3 mm² (−44.0 to 54.6) after proprioceptive exercise, and −11.2 mm² (−69.2 to 46.7) after control; diagonal mobilization differed from control by +133.5 mm² (95% CI 33.9 to 233.2; within-endpoint Holm-adjusted p = 0.037; dz = 0.74). In single-leg stance with eyes closed, COP surface asymmetry showed a nominal condition effect (F_(2,28)_ = 5.05, p = 0.013, ηp² = 0.265), with proprioceptive exercise reducing asymmetry relative to control (mean difference in change = −25.5 percentage points, 95% CI −45.3 to −5.7; within-endpoint Holm-adjusted p = 0.046; dz = 0.71). Complementary left heel and left forefoot load variables also showed nominal condition effects (both F_(2,28)_ = 4.31, p = 0.023, ηp² = 0.236), reflecting the same posterior–anterior load shift after proprioceptive exercise versus control. Nevertheless, none of these force-plate endpoints survived battery-wise Holm adjustment (adjusted p values 0.428 to 0.697).

## Discussion

The present randomized crossover trial indicates that, in youth male soccer players, a single session of diagonal mobilization or proprioceptive exercise did not produce broad acute improvements across the combined static and dynamic balance battery. In bipodal force-plate testing, outcomes were largely unchanged across conditions. The only nominal between-condition signal was a larger eyes-closed right-foot center-of-pressure (COP) surface area after mobilization than after control, a pattern more compatible with greater sway than with improved stability. However, the isolated increase in eyes-closed right-foot COP surface area after diagonal mobilization should not be interpreted as a beneficial balance response, because greater COP excursion or sway area during quiet stance is generally interpreted as reduced postural steadiness rather than improved stability. In eyes-closed single-leg stance, proprioceptive exercise was associated with reduced COP surface asymmetry relative to control, but this finding was isolated within a broader set of largely null force-plate results. By contrast, the clearest interpretable between-condition effect was observed for the principal dynamic-balance endpoint, the dominant-limb Y-Balance composite, for which mobilization attenuated the post-session decline seen after control. These results support a cautious interpretation centered on limited, task-specific acute responses rather than consistent enhancement of postural control. From an interpretive perspective, isolated nominally significant effects within a broad balance battery should be weighted less heavily than the predominance of unchanged variables, particularly when the protocol includes exploratory COP-derived outcomes that are sensitive to methodological variability. The present findings should however be interpreted as short-term responses in surrogate balance measures within a crossover design, not as direct evidence that either intervention reduces injury risk in youth soccer players.

The bipodal eyes-closed COP result after mobilization should not be interpreted as positive. A larger COP surface area indicates greater sway, so the observed increase is more compatible with a destabilizing response, an arousal-related fluctuation, or ordinary measurement noise than with improved balance. By contrast, the reduction in single-leg COP asymmetry after the proprioceptive session and the preservation of the dominant-limb Y-Balance composite after the active conditions may reflect genuine short-term neuromuscular responses, however, those signals were not replicated across a broader set of static and dynamic outcomes. Force-plate derived postural sway metrics in children and adolescents have shown acceptable test–retest reliability but limited responsiveness to low-dose or single-session balance interventions, which may explain why bipedal stance was relatively insensitive to brief manipulative input in our sample (Quatman-Yates et al., 2013; Barozzi et al., 2014). In contrast to our findings, Studnicki et al. (2025) reported significant time × group interactions for center-of-pressure parameters in quiet single-leg stance and landing tasks following a single session of diagonal mobilization compared with placebo in youth soccer players, suggesting that this manual therapy paradigm can acutely influence static and transitional balance under more challenging conditions. The absence of clear bipodal effects in our trial may therefore reflect both a ceiling effect of quiet bipedal stance in trained youth players and the fact that the mobilization was integrated into a broader warm-up rather than delivered as a standalone intervention targeting postural tasks with high stability demands.

Single-leg stance on the force plate showed somewhat larger pre–post variations than bipodal stance, yet these changes were small and not systematically greater after diagonal mobilization than after proprioceptive or control warm-ups, and no consistent reduction in inter-limb asymmetry was detected. Static and unstable unilateral postural control measures have previously been associated with prospective non-contact lower-extremity injury risk in elite youth soccer players, where greater sway and poorer stability identified athletes at higher risk (Kolodziej et al., 2021). Furthermore, observational work in professional and youth soccer has revealed meaningful correlations between static and dynamic balance abilities, reinforcing the notion that unilateral postural control is functionally relevant for soccer performance (Pau et al., 2015). Experimental studies on fatigue in young soccer players have shown that sport-specific exertion can acutely impair postural stability in single-leg tasks, underscoring the sensitivity of unilateral balance to neuromuscular state (Pau et al., 2014). Our trivial acute effects across all conditions suggest that a single brief exposure (whether manual mobilization or proprioceptive exercise) may be insufficient to induce measurable improvements in static unilateral postural control beyond normal test variability, particularly in already trained youth players.

Regarding dynamic balance, we found small, non-systematic increases in normalized Y-Balance Test reach distances and composite scores from pre- to post-intervention across all three conditions, without evidence that diagonal mobilization produced superior acute improvements relative to proprioceptive or control warm-ups, and without meaningful modification of inter-limb asymmetries. The YBT-LQ has been shown to provide reliable dynamic balance measures in youth athletes and is widely used as a field-based screening tool for directional balance deficits and injury risk (Calatayud et al., 2015; Linek et al., 2017). Moreover, limb dominance does not appear to meaningfully affect YBT performance in healthy youth, which aligns with the relatively symmetrical reach profiles we observed between dominant and non-dominant limbs (Stoddard et al., 2022). In contrast to our acute, single-session design, longer-term balance and proprioceptive interventions in youth soccer players have showed moderate-to-large improvements in dynamic balance and technical skills after 6–8 weeks of structured training programmes, including proprioceptive or balance-based warm-ups (Gidu et al., 2022; Mitrousis et al., 2023). In healthy adolescents, reported MDC95 values for YBT-LQ outcomes are approximately 4.90–16.10%, and in early adolescent cohorts composite-score MDC values of roughly 2–4% have been described, indicating that small post-session differences may fall within measurement error rather than reflecting a clearly meaningful functional improvement (Schwiertz et al., 2019). Thus, our small, non-differential acute changes in YBT performance primarily reflect short-term warm-up and familiarization effects rather than a specific neuromechanical advantage of diagonal mobilization.

The current findings should be interpreted in light of several limitations and their implications for practice. A limitation is that the proprioceptive condition lasted approximately twice as long as the diagonal mobilization and sham conditions. This dose/time mismatch introduces a potential confound related to exposure duration, attention, and physiological activation, so observed differences cannot be attributed exclusively to intervention type. Moreover, our design focused exclusively on acute responses to single exposures and did not assess medium- or long-term adaptations, whereas the injury-prevention and performance benefits of neuromuscular and proprioceptive training in youth sport typically emerge after sustained multi-week implementation. Moreover, all participants were male youth soccer players from a single competitive level and team, which limits generalizability to female athletes, younger age groups, or other team sports. Blinding should also be considered incomplete. Although a sham manual-contact condition was used, the treating therapist could not be blinded, and no post-session credibility or expectancy assessment was performed. Therefore, the extent to which participant beliefs about treatment assignment influenced performance cannot be determined. The use of a standardized pre-session warm-up may also have reduced the detectability of small intervention-specific acute effects. In addition, baseline fatigue/readiness was not formally quantified, so residual variation in acute physiological state across sessions cannot be excluded. Only one single-leg trial per visual condition and limb was collected, which likely increased measurement error and reduced reliability for COP-based outcomes. This limitation is important when interpreting isolated statistically significant findings within the single-leg force-plate battery.

Also, although force-plate and YBT measures used in this study are reliable and widely employed in youth populations, they capture only selected aspects of neuromuscular control and do not directly quantify game-specific performance or real-world injury outcomes. Future research should examine repeated applications of diagonal mobilization integrated into comprehensive neuromuscular training programmes, evaluate dose–response relationships, and explore whether combining manual therapy with targeted balance and strength exercises yields additive or synergistic effects on dynamic stability and injury risk in youth soccer. From a practical perspective, our results suggest that a single session of diagonal mobilization, does not produce practical relevant acute improvements in static or dynamic balance beyond those achievable with proprioceptive or control routines, and that any meaningful benefits of such interventions are likely to depend on sufficient training volume and long-term integration into evidence-based neuromuscular warm-up strategies.

## Conclusions

In youth male soccer players, a single session of diagonal hip and sacroiliac mobilization or a brief proprioceptive exercise sequence did not yield consistent, generalized acute improvements across bipodal stance, single-leg stance, and Y-Balance outcomes. The only between-condition result that remained statistically significant after within-outcome adjustment was preservation of the dominant-limb Y-Balance composite after mobilization versus control, whereas the force-plate findings were isolated exploratory signals of uncertain practical importance. These data do not support claims of meaningful acute balance enhancement or injury-prevention benefit from brief, stand-alone application of either strategy in already trained youth players. Rather, the findings should be interpreted as exploratory and hypothesis-generating, warranting replication in adequately powered studies with prespecified outcome hierarchies, duration-matched comparators, and crossover analyses that explicitly model period and sequence effects.

## Data Availability

The original contributions presented in the study are included in the article/supplementary material. Further inquiries can be directed to the corresponding author/s.
